# The Silent Spillover Threat: Nipah Virus Epidemiology, Pathogenesis, Clinical Manifestations, and Advances in Therapeutics and Vaccine Development

**DOI:** 10.3390/microorganisms14051109

**Published:** 2026-05-13

**Authors:** Elli-Panagiota Magklara, Maria Kkirgia, Andreas G. Tsantes, Petros Ioannou, Alexandra Mpakosi, Vasiliki Mougiou, Zoi Iliodromiti, Theodora Boutsikou, Nicoletta Iacovidou, Rozeta Sokou

**Affiliations:** 1 Neonatal Department, Aretaieio Hospital, National and Kapodistrian University of Athens, 11528 Athens, Greece; ellimaglara99@gmail.com (E.-P.M.); kirgiamaria@icloud.com (M.K.); vasiamoug@med.uoa.gr (V.M.); ziliodromiti@med.uoa.gr (Z.I.); theobtsk@med.uoa.gr (T.B.); niakobid@med.uoa.gr (N.I.); 2 Laboratory of Haematology and Blood Bank Unit, School of Medicine, “Attiko” Hospital, National and Kapodistrian University of Athens, 12462 Athens, Greece; andreas.tsantes@yahoo.com; 3 Microbiology Department, “Saint Savvas” Oncology Hospital, 11522 Athens, Greece; 4 School of Medicine, University of Crete, 71003 Heraklion, Greece; 5 Department of Immunology, General Hospital of Nikaia “Agios Panteleimon”, 18454 Piraeus, Greece; ampakosi@auth.gr

**Keywords:** Nipah virus, disease outbreaks, emerging pathogen, viral encephalitis, antivirals, vaccine development

## Abstract

Nipah virus (NiV) is an animal-borne RNA virus of the genus Henipavirus that poses a significant global health threat. This threat is driven by the virus’s high mortality rate, its capacity to cause epidemics, and the lack of licensed therapeutic interventions or vaccines. Since its initial identification during the 1998–1999 outbreak in Malaysia and Singapore, recurrent episodes have occurred primarily in Bangladesh and India, with mortality rates frequently exceeding 70%. Fruit bats of the genus Pteropus serve as the biological host for the virus. Transmission to humans occurs via contact with infected wildlife, consumption of contaminated products, such as freshly harvested date palm sap, or direct person-to-person exposure. Other modes of transmission, such as transplacentally or via breast milk, are still under investigation. The clinical presentation of NiV infection varies widely, from mild flu-like symptoms to life-threatening respiratory disease and acute encephalitis. It frequently attacks the nervous system, which can lead to coma, permanent neurological damage, or relapsing encephalitis. The virus enters host cells via ephrin-B2/B3 receptors, enabling systemic dissemination and infiltration of the central nervous system. Diagnosis relies primarily on RT-PCR and serological assays, and virus isolation requires high-containment laboratories. Management remains largely supportive, as no approved antiviral therapy exists. Experimental agents, such as remdesivir, favipiravir, and monoclonal antibodies such as m102.4, have shown promise in preclinical studies. Multiple vaccine platforms—including subunit, viral vector, mRNA, and nanoparticle-based approaches—are under development, though none is yet licensed for human use. Strengthened surveillance, infection control measures, and continued research are essential to mitigate the threat posed by this emerging pathogen. This review summarizes current knowledge on NiV, including its virology, epidemiology, pathogenesis, transmission, and recent progress in therapeutic and vaccine development.

## 1. Introduction

Nipah virus (NiV) is an animal-borne virus associated with severe impact on human health, classified within the order *Mononegavirales*, family *Paramyxoviridae*, subfamily *Paramyxovirinae*, and genus *Henipavirus* [1]. More than 60% of etiological agents implicated in human infectious diseases are zoonotic. In addition, RNA viruses are currently considered the primary etiological agents in approximately 44% of existing infectious diseases as they have an increased capacity to cross the species barriers, due to their brief generation circles and rapid evolutionary rates driven by high mutation frequencies [2].

NiV infection may result in severe pulmonary illness and encephalitis [3]. Considering its capacity to cause a pandemic, its extensive geographic distribution, and its high case fatality rate (CFR), the World Health Organization has included NiV in its list of priority pathogens [4]. Moreover, the Centers for Disease Control (CDC) and Prevention and the National Institute of Allergy and Infectious Diseases (NIAID) classify NiV as a potential bioterrorism agent [5].

This review offers a comprehensive evaluation of NiV, examining its structural characteristics, replication strategies, epidemiology, and latest advancement in therapeutic interventions and vaccine development. It also analyzes available data on pathogenesis, modes of transmission, and specific considerations related to pregnancy and neonatal and pediatric populations.

## 2. Methods

A narrative literature review was conducted to synthesize current knowledge on Nipah virus. PubMed, Google Scholar, and Web of Science were searched up to February 2026 using terms including “Nipah virus,” “epidemiology,” “pathogenesis,” “clinical manifestations,” “encephalitis,” “diagnosis,” “treatment,” and “vaccines.” Clinical trial information was retrieved from ClinicalTrials.gov, while outbreak data and epidemiological updates were obtained from the World Health Organization and the Centers for Disease Control and Prevention. Vaccine development progress was reviewed using resources from the Coalition for Epidemic Preparedness Innovations and the WHO R&D Blueprint. Peer-reviewed articles, systematic reviews, case reports, and clinical guidelines published in English were included. Preclinical studies evaluating therapeutic agents and vaccine candidates were included when human data were limited or unavailable. Although emphasis was placed on publications from the last decade, earlier foundational studies were also incorporated.

## 3. History and Epidemiology

NiV exhibits an intricate epidemiological footprint, marked by occasional incidences, shifting modes of spread and elevated mortality rates. Malaysia was the site of the first documented human NiV infection, which occurred between 1998 and 1999, during an outbreak of neurological and respiratory illness on pig farms that resulted in 265 human infections and 105 deaths [6,7]. The disease was misattributed to Japanese encephalitis virus (JEV). However, the absence of the characteristic brain lesions on neuroimaging typically associated with JEV- such as bilateral thalamic lesions and basal ganglia involvement, along with negative laboratory results for JEV, led investigators to conclude that a previously unidentified virus was responsible for the infection [8]. In 1999, the etiologic agent was isolated and identified as a new paramyxovirus. NiV derives its name from Sungai Nipah village in Negeri Sembilan, the site of the first viral isolates [9]. The outbreak later extended to Singapore through the trade of pigs carrying the virus, leading 11 slaughterhouse workers to develop symptoms and resulting in one death [10].

Researchers have categorized human NiV infections into two primary variants: the Malaysian strain (NiV-MY), responsible for the 1998–1999 surges in Malaysia and Singapore and the 2014 outbreak in the Philippines, with 17 cases and 9 deaths [11], and the Bangladesh strain (NiV-BD), which is linked to more severe clinical manifestation and higher mortality rates [12]. Their genomes share a 91.8% genetic similarity. Despite this high level of homology, the NiV-B genome is slightly larger, containing six additional nucleotides compared to the NiV-M strain [13].

The Malaysia strain is characterized by a lower case fatality rate of approximately 40% and presents primarily as acute encephalitis, with transmission historically linked to intermediate pig hosts rather than direct person-to-person spread [14].

In contrast, the Bangladesh strain—and its closely related Indian variants—is significantly more lethal, with mortality rates often exceeding 70%. Epidemiologically, NiV-B is notorious for its capacity for human-to-human transmission and its dual clinical presentation, where patients frequently suffer from both severe neurological inflammation and acute respiratory distress syndrome (ARDS) [13].

The NiV-B genotype has further diverged into two distinct sub-lineages, known as NiV-BD1 and NiV-BD2. NiV-BD1 was the first to be identified during the 2004 outbreaks and is primarily localized in the northern and central regions, whereas the NiV-BD2 emerged more prominently after 2008 and exhibits a broader geographic distribution, spreading into the southern regions of the country [15].

While both sub-lineages share a high overall mortality rate exceeding 80%, clinical observations suggest that NiV-BD2 may be associated with a higher prevalence of respiratory distress, whereas cases of NiV-BD1 have been noted for slightly longer median durations of hospitalization [15,16].

Since 2001, Bangladesh has been affected by consecutive NiV outbreaks and the majority of cases have been observed in 4 administrative divisions: Dhaka, Khulna, Rajshahi, and Rangpur [17,18] 13 May 2026 12:03:00 p.m. From 2001 onwards, Bangladesh has reported 348 cases of NiV infection and 250 deaths, yielding an overall case fatality rate of 72%. A significant portion close to 50% (*n* = 162) were classified as primary infections associated with intake of unprocessed date palm sap and 29% were attributed to direct transmission between individuals.

The most recent confirmed case of NiV infection, which was fatal, involved a female patient and occurred on 3 February 2026 [19]. The earliest recorded NiV outbreak occurred in Siliguri, West Bengal, in 2001, with 66 cases and 49 deaths, corresponding to a case fatality rate of 74%, followed by later outbreaks in 2007 (5 cases, 5 deaths, CFR 100%), 2018 (23 cases, 21 deaths, CFR 91%), 2019 (1 case, 0 deaths, 0% CFR), 2021 (1 case, 1 death, CFR 100%), 2023 (6 cases, 2 deaths, CFR 33%), 2024 (2 cases, 2 deaths, CFR 100%) and in 2025 (5 case, 2 death, CFR 40%) [6,20,21]. As of February 2026, 2 cases of NiV infection have been confirmed in West Bengal, India; the two subjects were nursing professionals (one female and one male), employed at the same private medical facility and had physical contact with each other. Both patients developed symptoms reflective of severe NiV infection and were hospitalized. The second patient showed clinical improvement, whereas the first remained in critical condition [20,22].

## 4. Genome of NiV

**Figure 1:**
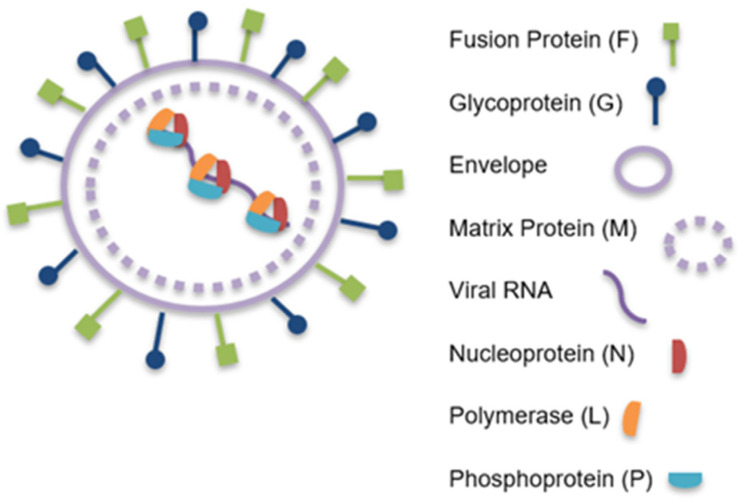
Representation of the Nipah virus genome.

Nipah virus (NiV) is a member of the family *Paramyxoviridae* with a single-stranded, negative-sense RNA genome. It belongs to the genus Henipavirus, alongside Hendra virus (HeV). The NiV genome follows the canonical paramyxovirus gene order 3′-N-P-M-F-G-L-5′ [23] and it encodes 6 structural proteins, including the nucleocapsid (N), phosphoprotein (P), matrix (M), fusion (F), attachment glycoprotein (G), and large (L) polymerase and 3 accessory proteins (C, W, and V), which are encoded within the P gene, along with the P protein (Figure 1). Compared with most paramyxoviruses, NiV possesses a larger genome (18,246 nucleotides), primarily due to extended noncoding regions and the larger size of its P gene [5,23]. The viral ribonucleoprotein (vRNP) complex—assembled from the N, P, and L proteins in conjunction with the genomic RNA—serves as the primary engine for RNA synthesis. The viral M protein is fundamental for virion morphogenesis and budding [24]. The envelope glycoproteins of NiV, the attachment glycoprotein (NiV-G) and the fusion protein (NiV-F), are the engine for cellular penetration and formation of syncytia [25,26]. Along with the accessory (non-structural) proteins C, W, and V, the F and G proteins contribute to the enclosure of NiV genetic material [27].

The Nipah virus N protein unites with the P and L proteins to construct ribonucleoproteins, structures that wrap the viral RNA genome in a strict arrangement of one N protein for every six nucleotides. These RNPs serve as the essential machinery for the primary transcription of viral messenger RNAs [28].

One of the most significant recent discoveries regarding the N protein is its ability to drive the formation of Inclusion Bodies (liquid-like droplets) in coordination with the P protein, through a process known as liquid–liquid phase separation. These droplets act as “viral factories,” concentrating all the necessary tools to speed up replication [29].

The N gene is the primary biomarker prioritized for molecular diagnostic tests, due to its high level of sequence conservation and abundant expression during active infection [30].

P protein anchors the viral polymerase directly onto the nucleocapsid, allowing replication and transcription of the virus. The V and W proteins arise through transcriptional RNA editing, whereas the C protein is produced from an alternative open reading frame situated downstream of the P protein’s initiation site. Each of the four proteins derived from the P gene demonstrate the ability to actively suppress the host’s interferon immune response [28,31]. The P gene is a promising diagnostic tool. As it undergoes mRNA editing to produce V and W proteins, targeting conserved regions of this gene allows for the detection of multiple viral transcripts in a single test, which is useful for verifying active viral replication.

The L protein handles several distinct enzymatic tasks, operating entirely within the host cell’s cytoplasm. Its enzymatic activity is driven by three catalytic domains—the RNA-dependent RNA polymerase (RdRp), the polyribonucleotidyl transferase (PRNTase), and the methyltransferase (MTase). Throughout the transcription process, it is responsible for the synchronized synthesis of messenger RNA, ensuring each strand is modified with a protective 5′ cap and a 3′ polyadenylated tail [32].

The F protein t is synthesized as an inactive precursor, F0, and must be cleaved into two subunits (F1 and F2) to become fusion-competent. F0 is synthesized and travels to the cell surface in its inactive form and then it is pulled back inside the cell via clathrin-mediated endocytosis, triggered by a specific signal in its cytoplasmic tail. Once inside the endosome, it is cleaved by host proteases—specifically Cathepsin L or Cathepsin B. The now-active, cleaved F protein is recycled back to the plasma membrane to be incorporated into new virus particles [33].

The G protein is a type II transmembrane glycoprotein that serves as an attachment factor that initiates infection by latching onto specific surface proteins on the host cell, specifically ephrin-B2 and ephrin-B3, which act as the virus’s primary gateways for entry [28,34]. The G protein is the primary target for identifying long-term immunity and distinguishing between different Henipaviruses. Because G is a surface protein, it triggers a strong antibody response in the host [35].

The viral M protein attaches to the interior of the cell’s plasma membrane, acting as a structural link between the internal ribonucleoprotein and the external surface glycoproteins. Although the Nipah virus reproduces its genetic material solely within the cytoplasm, the matrix protein follows a transport path that involves an essential transition through the cell nucleus to achieve its active state through ubiquitination [36].

Genome sequencing has detected at least 2 discrete strains of NiV associated with outbreaks in different geographic regions, i.e., NiV Bangladesh and NiV Malaysi [37]. Although these strains share a genetic similarity of ~91.8%, several differences were documented regarding mortality rate, incubation period and clinical symptoms [38]. Sun et al. [39] identified two clades—the Bangladesh clade, encompassing strains from Bangladesh, India, and Thailand, and the Malaysia clade, which includes strains from Malaysia, Thailand, and Cambodia. Phylogeographic evidence suggests that the virus’s latest common progenitor dates back to approximately 1853. The Malaysian clade is estimated to have emerged around 1960, while the Bangladesh clade likely arose around 1971 [39].

## 5. Pathogenesis

The virus invades the host via the oral and nasal mucosa, leading to infection [40]. The envelope-associated F and G glycoproteins of NiV mediate host cell attachment and viral entry. NiV G glycoprotein binds to the cellular receptor ephrin-B2, which is detected on nerve cells, vascular smooth muscle, and endothelial cells of small arteries [41]. Ephrin-B3, a related molecule with diminished binding strength for NiV G, was identified as a functional alternative receptor for both NiV and HeV, though HeV demonstrates lower binding affinity to ephrin-B3 compared with NiV [42].

**Figure 2:**
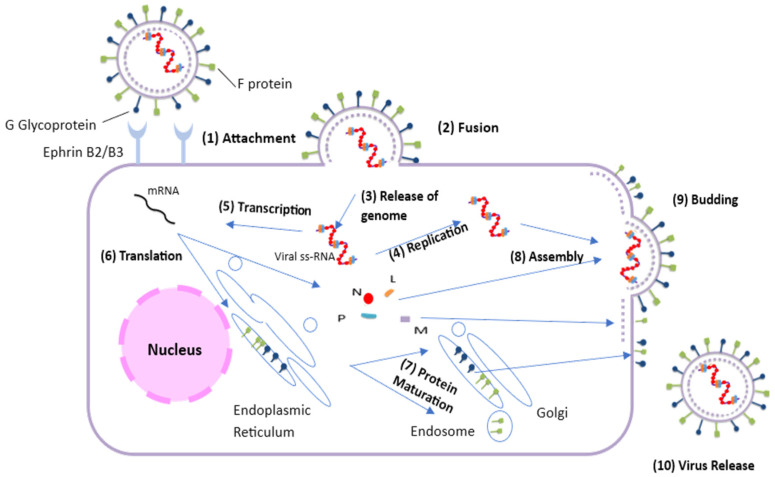
Replication cycle of Nipah Virus. The virus enters the body via the oronasal route and binds to ephrin-B2/B3 receptors via its G glycoprotein. Activation of the F protein enables membrane fusion and the release of the viral genome into the cytoplasm, where replication occurs. Newly formed virions assemble and bud from the cell, spread via the bloodstream, and can infect multiple organs, including the brain.

The entry process begins when the NiV G protein anchors to host ephrin-B2 or -B3 receptors. This binding event mediates structural changes in the F protein, leading to fusion of the viral and cellular membranes and subsequent delivery of the viral ribonucleocapsid into the intracellular environment [43,44]. Following entry, the viral polymerase triggers transcription at the genomic promoter [23,45,46]. While most viral glycoproteins are processed through the standard ER-to-Golgi pathway, the F glycoprotein, undergoes processing and maturation from the ER to the Golgi apparatus and then in the endosomal compartment [47,48]. The cytoplasmic tails of the F and G glycoproteins interact with the M protein, which orchestrates viral morphogenesis and exit at the cell membrane [49,50,51] (Figure 2).

Human infection with NiV is primarily initiated via the oronasal route following direct exposure to infected excretions or ingestion of contaminated food products, with the lining of the bronchi and type II pneumocytes serving as the principal target cells [1]. Infection of the respiratory epithelium by NiV incites a robust inflammatory cascade marked by the systemic release of proinflammatory signaling molecules, including interleukin (IL)-1α, IL-6, IL-8, and granulocyte colony-stimulating factor (G-CSF) [52]. At the same time, viral particles can enter the bloodstream and disseminate systemically either in a cell-free form or bound to host leukocytes At the same time, viral particles can enter the bloodstream and disseminate systemically either in a cell-free form or associated with host leukocytes, facilitating immune evasion and “Trojan horse”-like dissemination [53]. Consequently, NiV can involve organs such as the spleen and kidneys, leading to organ failure [54]. Furthermore, it has the ability to enter into the central nervous system through two pathways, by disrupting the blood-brain barrier, enabling direct infection of neurons and microglial cells and through the olfactory nerve [55].

## 6. Transmission

NiV transmission involves reservoir and intermediate hosts, as well as direct human-to-human transmission [56]. The main reservoir of NiV is *Pteropoid* fruit bats, also known as flying foxes [57], whose infection appears to be asymptomatic. They are attracted by the fruits and they often feed on and contaminate date palm sap with their oral secretions, urinary excretions, and fecal matter [1]. The virus can remain viable for days in solutions with high sugar concentrations, such as fruit mash [58]. Transmission occurs through direct exposure to infected animals or through consumption of food or fruit contaminated with excretions from infected reservoir or intermediate hosts, including bats and pigs [56].

Pigs act as intermediate hosts with transmission to humans occurring mainly through close contact with infected animals or their tissues during handling and slaughter. This transmission pattern was especially evident during outbreaks in Malaysia and Singapore, whereas in Bangladesh, the predominant mode of viral spread was through the consumption of contaminated date palm sap [1]. Furthermore, human-to-human transmission of NiV may occur following close contact with infected patients. Clinical practitioners are more at risk of NiV infection through direct exposure to contaminated respiratory droplets or other body fluids from patients. Such an outbreak was documented in India in 2001, emphasizing NiV’s potential for nosocomial transmission [59].

Transplacental transmission is another possible route currently under investigation. To date, there is no report of transplacental transmission of NiV in humans, however studies on experimentally infected animals (cats, horses, pigs) demonstrated that the virus can cross the placenta, resulting in high viral loads in the placenta, uterine fluid, and fetal tissues [60]. In addition to possible in utero transmission, postnatal spread of NiV has also been considered. A 2023 study conducted in Bangladesh detected NiV RNA in human breast milk, raising concerns regarding potential breastfeeding-associated transmission [61].

**Figure 3:**
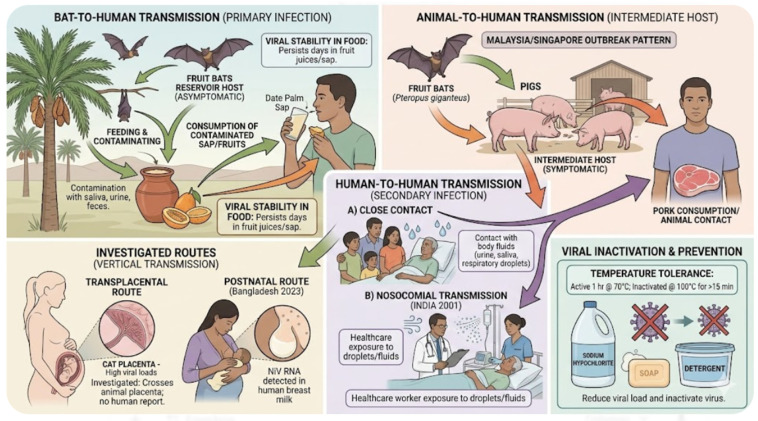
Transmission of Nipah Virus. Colored arrows indicate the direction of viral transmission: green for primary bat-to-human routes, orange for intermediate animal hosts, and purple for human-to-human secondary infection. (This figure was created with Google Gemini 3 Flash).

NiV exhibits significant environmental resilience at 22 °C, maintaining infectivity for 3 days in fruit juices and for a full week in model date palm sap. While the virus remains active at 70 °C for up to 60 min in high sugar content solution (although a significant decrease in its concentration will occur), it is completely destroyed by 15 min exposure to 100 °C [24]. Standard decontamination measures and the application of potent germicides can reduce the viral load and inactivate the virus [1,62]. (Figure 3).

## 7. Clinical Manifestations

NiV infection presents with a wide clinical spectrum, ranging from mild, nonspecific illness to rapidly progressive disease that is often fatal [63]. The infection primarily affects the central nervous system and the respiratory tract, although the severity and clinical presentation vary depending on the viral strain and geographic region [64].

### 7.1. Incubation Period and Onset

The incubation period of NiV infection most commonly ranges from 3 to 14 days, although wider variability has been reported. Several outbreaks documented incubation periods extending from several weeks to months [65].

### 7.2. Prodromal and Early Symptoms

The initial phase of NiV infection is characterized by nonspecific, flu-like symptoms [66], which may complicate early diagnosis. Common prodromal features include fever, which is reported in nearly all symptomatic cases, along with headache, myalgia, fatigue or lethargy, dizziness or vertigo, nausea and vomiting, sore throat or other upper respiratory symptoms. These early manifestations are often indistinguishable from other viral febrile illnesses [63,67,68].

### 7.3. Neurological Manifestations

Neurological involvement is a dominant feature of NiV infection and often indicates disease progression and a severe clinical course. Acute encephalitis typically develops within days of initial symptom onset and may deteriorate rapidly [69]. A recent systematic review [69] including 504 patients reported neurological involvement in more than 90% of confirmed cases. The most frequently observed neurological symptoms were headache (63.9%) and depressed level of consciousness (63.5%), followed by somnolence (44.0%) and seizures (21.6%). Early manifestations often include altered mental status, confusion, and disorientation, which may progress to profound drowsiness and coma within 24–48 h. Frequently observed neurological signs include pupillary abnormalities (54.0%), hyporeflexia (44.5%), cranial neuropathies (36.0%), and segmental myoclonus (31.0%). Additional findings include hypotonia, areflexia, focal neurological deficits, gaze palsy, ptosis and cerebellar signs such as ataxia, gait instability and brainstem dysfunction [69]. In severe cases, autonomic instability, including hypertension and tachycardia, may also occur [63]. Neuroimaging findings demonstrate multifocal lesions involving both gray and white matter. Brainstem hyperintensities represent the most frequent MRI abnormality, followed by cortical and deep white matter lesions, findings that support the marked neurotropism of the virus [69]. Clinical observations from the Malaysian outbreak further demonstrated that neurological deterioration can occur rapidly, with some patients progressing to seizures and coma within days of hospitalization. Neurological involvement in NiV infection is diagnosed through an integrated evaluation of clinical manifestations characterized by encephalitis, altered level of consciousness, and evidence of brainstem dysfunction, neuroimaging findings, and laboratory confirmation. Magnetic resonance imaging may reveal characteristic lesions, predominantly involving white matter. Definitive diagnosis is established by molecular detection (RT-PCR) and/or serological assays, whereas case definition criteria require the concurrence of a compatible clinical syndrome and a relevant epidemiological exposure history [69].

### 7.4. Respiratory Involvement

Respiratory manifestations vary significantly across outbreaks and viral genotypes. They are more prominent in Bangladesh and India compared with Malaysia and Singapore [70]. Respiratory features include cough, dyspnea or difficulty in breathing, atypical pneumonia and acute respiratory distress syndrome (ARDS) [71]. Severe respiratory involvement is reported in up to 75% of cases in certain outbreaks and is strongly associated with increased mortality and person-to-person transmission [68].

### 7.5. Systemic Features

Beyond respiratory and neurological involvement, NiV infection may present with additional systemic complications, particularly in severe cases.

Gastrointestinal manifestations include vomiting and diarrhea during the early phase of illness, while more severe cases may develop gastrointestinal bleeding [24]. These findings are consistent with systemic disease progression and endothelial involvement.

Septicemia has been described in advanced stages of infection and may coexist with multiorgan impairment. Renal involvement can occur, ranging from functional impairment to renal failure [52].

Cardiovascular involvement is increasingly recognized as a significant contributor to disease severity. Myocarditis, characterized by myocardial inflammation, is also reported and may result in myocardial dysfunction, arrhythmias, and heart failure [72].

Systemic endothelial infection and dysfunction play a central role in these manifestations. Viral-induced endotheliitis and vascular injury may predispose to disseminated intravascular coagulation (DIC), contributing to thrombotic occlusion, impaired organ perfusion, and progression to multiple organ dysfunction syndrome (MODS). Occasional vasculitic involvement of mesenteric and pancreatic vessels provides a pathophysiological basis for rare reports of pancreatitis in severe disease [72,73].

Overall, these extrapulmonary and extra-neurological manifestations reflect hematogenous viral dissemination and diffuse small-vessel vasculitis, ultimately contributing to MODS in advanced infection [52].

### 7.6. Asymptomatic Infection

The frequency of asymptomatic infection varies widely by outbreak. Asymptomatic or subclinical cases were documented primarily in Malaysia and Singapore, with reported rates ranging from 8% to over 40%. In contrast, asymptomatic infection is rare or absent in Bangladesh and India, where disease presentation tends to be more severe [74,75,76].

### 7.7. Relapse, Late-Onset Disease and Long-Term Complications

A distinctive feature of NiV infection is the occurrence of relapsing or late-onset encephalitis, which may develop months to years after apparent recovery [77,78]. Clinical manifestations during relapse resemble those observed in acute encephalitis and commonly include seizures, focal neurological deficits, altered consciousness, and new-onset behavioral or cognitive disturbances [73]. Compared with acute NiV, patients with relapsed or late-onset disease are less likely to present with fever, coma, or brainstem signs, but more frequently exhibit seizures and focal cortical deficits, consistent with relatively localized cerebral involvement on neuroimaging.

In the Malaysian cohort described by Tan et al. [77], relapsed or late-onset encephalitis occurred in approximately 7.5% of survivors of acute encephalitis and in 3.4% of patients with previously non-encephalitic or asymptomatic infection. Mortality during relapsed and late-onset encephalitis was reported at 18%, lower than the 40% mortality observed in acute disease. However, survivors of relapsed disease experienced worse long-term neurological outcomes, with residual deficits reported in 61% of cases compared with 22% following acute encephalitis [77].

Long-term neurological and psychiatric sequelae are also well documented among survivors of acute NiV encephalitis, particularly depression [79]. Cohort studies suggest that approximately 20–30% of survivors develop persistent complications. These include epilepsy, chronic fatigue, cognitive impairment, memory deficits, depression, personality changes, motor weakness, cranial neuropathies, and parkinsonian features [78,79]. These findings highlight that NiV is associated not only with high immediate mortality but also with substantial long-term neurological and neuropsychiatric morbidity.

## 8. Pediatric Population

Current evidence indicates that the clinical manifestations and management of NiV infection in pediatric patients are broadly similar to those in adults. Infection is characterized by acute onset of fever, headache, confusion, and neurological symptoms that may rapidly progress to encephalitis. Respiratory symptoms, such as cough and shortness of breath, are frequently observed, with the potential for acute respiratory distress. Mortality is high, ranging from 40% to 75%, depending on the healthcare setting and disease severity [80,81].

All pediatric age groups are susceptible, but school-aged children and adolescents appear to have a higher frequency of severe disease and death, possibly due to increased exposure to contaminated food or care of infected patients. There are no approved antiviral drugs or vaccines, and treatment is limited to supportive care, including monitoring, management of encephalitis (seizure control, intracranial pressure management), respiratory support, and isolation to prevent transmission [5,81,82,83].

## 9. Pregnancy and Neonatal Considerations

Although NiV infection is associated with severe disease in adults, data on its impact in the neonatal period are limited. It is well established that infants and neonates are particularly susceptible to viral infections because their immune systems are not yet fully mature [84]. To date, no confirmed cases of congenital NiV infection in humans have been documented. However, recent evidence indicates that maternal humoral immunity against NiV can be transferred across the placenta; a neonate born to a survivor of NiV disease in Bangladesh tested negative for NiV RNA and IgM antibodies but exhibited high titres of anti-NiV IgG, consistent with passive placental transfer of maternal antibody without active neonatal infection [85]. In addition, a postpartum transmission scenario was reported during a NiV outbreak in Bangladesh in January 2023, raising concern for perinatal or postnatal transmission pathways. In this case, a 20-year-old woman developed acute NiV encephalitis approximately 14 days after consuming raw date palm sap, shortly after delivering her infant by cesarean section. Following a period of breastfeeding that ended when the mother was hospitalized, the newborn contracted NIV. The resulting severe respiratory illness proved fatal, leading to death. During the mother’s acute phase of illness, two separate samples tested positive for NiV RNA with the Reverse Transcription-Polymerase Chain Reaction (RT-PCR), representing the first documented detection of NiV genetic material in human breast milk. While detection of viral RNA does not confirm the presence of infectious virus, the absence of alternative exposures and the prolonged close contact between mother and infant suggest maternal-to-infant transmission as the most probable source of infection [61]. On the other hand, animal models provide biological plausibility for in utero exposure and NiV transmission. Mungall et al. provide the first experimental demonstration that NiV can be transmitted vertically in cats. In that study, NiV genomes and high virus loads were detected in the placenta, uterus, and fetal itself, demonstrating vertical transmission [60]. Given the limited available evidence, further research on NiV infection in pregnant women and neonates, along with clarification of potential transmission routes—including in utero, intrapartum, and via breastfeeding—is urgently needed.

## 10. Diagnosis

Because early symptoms of Nipah virus are largely non-specific, diagnosis can be difficult. However, early detection remains vital for improving patient survival rates and implementing the necessary containment measures to prevent a broader epidemic.

The hallmark of Nipah virus neurological involvement is a rapidly progressing acute encephalitis. Diagnostic suspicion is raised when a patient presents with a sudden decline in mental status, often transitioning from initial drowsiness to deep coma within 24 to 48 h. Classic clinical indicators include brainstem signs, such as abnormal pupillary reflexes, oculomotor palsy, and severe autonomic instability manifested by fluctuating blood pressure and tachycardia [69]. In many cases, the virus also affects the spinal cord or peripheral nerves, resulting in areflexia or hyporeflexia, which distinguishes NiV from many other viral encephalitides that typically present with upper motor neuron signs [78].

Patients with NiV infection frequently present with specific laboratory anomalies, most notably thrombocytopenia and leukopenia. Hepatic involvement is common, with elevation of liver enzymes, while hyponatremia is the most significant electrolyte disturbance. Cerebrospinal fluid (CSF) analysis often reveals lymphocytic pleocytosis and elevated protein levels, consistent with standard viral meningitis profiles. Neuroimaging, specifically Magnetic Resonance Imaging, plays a pivotal role in the diagnosis of NiV. The findings are often considered “signature” markers that distinguish NiV from other types of viral encephalitis, such as Japanese Encephalitis, and include disseminated, small (2–7 mm) high-signal intensity lesions primarily localized within the subcortical and deep white matter [86].

A comprehensive diagnostic strategy for NiV infection requires integrating virus and antibody detection methods. Because these samples are highly infectious, they must be collected and handled carefully in high-containment labs. While initial isolation can occur in Biosafety level-3 (BSL-3) settings depending on national biosafety regulations (i.e., in Kerala in 2023 with the usage of mobile BSL-3 lab), full virus cultivation requires a BSL-4 laboratory [87]. However, chemical inactivation of the virus renders the samples safe to be analysed in a BSL-2 facility [88].

The optimal method for diagnosing NiV infections is polymerase chain reaction (PCR) due to its diagnostic accuracy. Samples that can be tested include nasal secretions, cerebrospinal fluid (CSF), urine, and blood. Several PCR-based assays were developed for NiV detection, including conventional reverse transcription (RT)-PCR, nested RT-PCR, and real-time RT-PCR. The latter method is the most frequently used, as it exhibits approximately 1000-fold greater sensitivity than conventional PCR. NiV RT-PCR tests were designed to target conserved N, M, or P gene segments. Wang et al. in their paper highlight that the first 12 nucleotides of the 5′ and 3′ genomic termini of the N gene are highly conserved and complementary across all isolates. Most molecular assays target the core of the N gene sequence because it is the most stable and heavily transcribed portion of the genome. They also state that diagnostic systems targeting the M gene often focus on the central portion of the gene, which is responsible for viral assembly and budding. As far as test systems developed for the P gene are concerned, they often focus on the conserved poly-G “stutter” site. This is the specific sequence where the polymerase inserts extra Gs to create the V and W proteins [89]. In 2018, a one-step qRT-PCR assay targeting the space between the F and G genes was developed, enabling accurate quantification of replicative NiV RNA and potentially offering greater precision than standard qRT-PCR [90]. Enzyme Linked Immuno Sorbent Assay (ELISA) is the predominant serological methodology for diagnosis owing to its high sensitivity, rapidity, simplicity, and safety [87]. Within 7 days of the first symptoms, IgM antibodies reach levels detectable by diagnostic testing [91]; in 50% of patients, they are present on day 1 and in all patients by day 3 after symptoms begin. IgG seroconversion is observed in 31% of patients within 48 h of symptom onset [30], with 100% seropositivity achieved by day 18; IgG levels persist for several months [92]. A landscape analysis of Mazzola et al., which provides a comprehensive review of the current diagnostic tools available and in development, identifies 13 commercial ELISA kits globally (primarily from manufacturers in the US, India, and Canada), available for research use only. Most available immunoassays focus on detecting the body’s immune response to the Nipah virus G protein, with specific tests available for IgM, IgG, and IgA antibodies. Additionally, certain manufacturers offer ELISA kits that target the nucleoprotein (NP) across all three antibody classes, while others provide general tests designed for broad Nipah antibody detection [93].

Immunohistochemistry represents an additional approach for diagnosing NiV infection that uses formalin-fixed tissues. Since replication of the virus occurs in the vascular endothelium, a variety of tissues are appropriate for use, including brain, lung, spleen and kidney [87].

Rapid screening modalities are increasingly critical for outbreak management. Several studies have documented the development of NiV diagnostic prototypes designed for point-of-care (POC) use. Near-POC platforms operate in basic laboratory environments with limited resources, while true POC platforms are intended for use in non-laboratory settings without specialized equipment or facilities [93]. One example of such a test is the Truenat Nipah POC system, a portable, battery-operated PCR platform, with high sensitivity and specificity [94]. Lateral flow assays (LFA) for NiV represent a novel addition to the Nipah diagnostics landscape and have the potential to transform screening at true POC settings; however, it is still under development. The Mazzola et al. paper outlines a variety of lateral flow technologies currently being adapted for Nipah virus detection, ranging from standard antigen LFAs that identify viral proteins like the nucleoprotein in swabs to antibody LFAs designed for rapid serological screening via finger-prick blood samples. Furthermore, the study highlights the emergence of molecular “hybrid” LFAs, which utilize simplified isothermal amplification methods to detect viral RNA while providing a user-friendly visual result on a traditional test strip instead of requiring complex laboratory instrumentation [93].

## 11. Prognostic Factors

Disease outcome is strongly influenced by viral strain characteristics and host-related factors. The Malaysian outbreak was associated with a mortality rate of approximately 40%, whereas outbreaks in Bangladesh and India have reported case fatality rates exceeding 70% [63,95].

Poor prognostic indicators include advanced age [73], early and severe neurological involvement—particularly seizures occurring within 24–48 h of symptom onset—early altered mental status, and reduced level of consciousness [63,69]. Respiratory involvement was also associated with increased severity and mortality in subsequent outbreaks [64,96]. Additional markers of poor outcome include abnormal pupillary responses, autonomic instability, hypertension, tachycardia, and systemic inflammatory manifestations [69].

Beyond neurological involvement, Chong et al. reported several systemic predictors of mortality during the Malaysian outbreak. These included diastolic hypertension, high-grade fever (≥40 °C), hyperglycemia, thrombocytopenia, and indicators of critical illness such as the need for inotropic support or mechanical ventilation. These findings highlight the contribution of severe systemic inflammation and hemodynamic instability to poor clinical outcomes. Although seizures and myoclonus carried increased mortality, their predictive value was less pronounced compared with the systemic parameters [97].

## 12. Treatment

### 12.1. General Management and Supportive Care

To date, no antiviral drugs have been approved for the treatment of NiV infection, and clinical management remains largely supportive [98]. This is particularly relevant for patients presenting with acute encephalitis syndrome and/or severe respiratory involvement. Supportive measures include airway protection, oxygen therapy or mechanical ventilation, anticonvulsant treatment, maintenance of fluid and electrolyte balance, prevention of venous thromboembolism, treatment of secondary bacterial infections, and rehabilitation of survivors. These measures remain the foundation of care across all outbreaks. Despite intensive supportive management, mortality remains high, especially in outbreaks caused by Bangladesh and Indian strains, highlighting the urgent need for effective targeted therapies [99,100].

### 12.2. Ribavirin and Other Repurposed Antivirals

Ribavirin is the most frequently used antiviral agent during NiV outbreaks due to its broad-spectrum antiviral activity and ability to cross the blood–brain barrier. In vitro studies have demonstrated inhibitory effects on NiV replication [101]. During the 1998–1999 Malaysian outbreak, an open-label trial suggested a reduction in mortality and the long-term neurological complications among ribavirin-treated patients [102]. However, this study was non-randomized, and later clinical use during outbreaks in India involved very small sample sizes, precluding definitive conclusions.

More recently, ribavirin was administered during the 2018 outbreak in Kerala, India [66]. Of the 12 confirmed cases, ribavirin was empirically administered to 6 patients, of whom 2 survived, while all 6 patients who did not receive ribavirin died. However, one of the survivors did not present with encephalitis. Among patients with NiV encephalitis treated with ribavirin, the fatality rate was 80%, corresponding to an estimated 20% reduction in mortality. This corresponded to lower observed mortality among treated patients than among those who did not receive ribavirin. However, the small sample size and lack of randomization precluded definitive conclusions regarding therapeutic efficacy. Ribavirin was also used as post-exposure prophylaxis in 8 healthcare workers during the same outbreak, none developed infection, although mild to moderate adverse effects were reported.

Alla et al., (2024) [73] reported that among 92 documented cases, 39 patients (42.3%) received active treatment, including pharmacological and interventional therapies. Ribavirin was administered to 5 patients (12.8%), and notably, 4 of the 7 patients who survived had received ribavirin. Most survivors were younger than 30 years, suggesting a possible interaction between host factors and therapeutic response. However, the small number of treated patients and the absence of controlled comparisons limit interpretation of these findings. Importantly, animal studies have not shown a survival benefit, demonstrating only delayed disease progression without prevention of death. Combined ribavirin–chloroquine therapy was also ineffective in hamster models [103]. As a result, the clinical efficacy of ribavirin remains uncertain, and its routine use is controversial despite recommendations from some national health authorities in the absence of alternatives [103,104]. Acyclovir was used empirically during the Singapore outbreak among abattoir workers, with favorable outcomes reported; however, it remains uncertain whether acyclovir contributed to these outcomes. Its use in suspected NiV infection reflects standard encephalitis management protocols rather than demonstrated antiviral activity against NiV [105]. To date, no in vitro studies or additional in vivo investigations evaluating acyclovir activity against NiV have been reported. Nevertheless, acyclovir is routinely administered in cases of suspected encephalitis to provide early empirical coverage for herpes simplex virus (HSV) encephalitis, a treatable condition in which prompt initiation of therapy is critical to reduce mortality and long-term neurological sequelae [9].

### 12.3. Experimental Antiviral Agents

Several antiviral compounds have demonstrated promise in preclinical studies, particularly when administered early. Remdesivir has shown the most compelling results to date, achieving 100% survival in African green monkey models when administered shortly after infection. Delayed administration markedly reduced efficacy [106]. Favipiravir, an RNA- dependent RNA polymerase inhibitor approved for influenza in Japan, provided complete protection in lethal hamster models when administered immediately post-infection and has demonstrated strong anti-Henipavirus activity [107]. Balapiravir (R1479), a nucleoside analog polymerase inhibitor, demonstrated in vitro activity against NiV and Hendra virus. However, its poor bioavailability and prior discontinuation in clinical trials for other viral infections due to toxicity and lack of efficacy limit its current clinical applicability. Nevertheless, 4′-modified nucleosides such as R1479 may serve as a structural basis for the development of broad-spectrum antiviral agents with activity across both positive- and negative-sense RNA virus families [108].

Poly(I)-poly(C12U), an interferon inducer, showed partial protection in animal models [109]. Soluble ephrin B2, the cellular receptor for the NiV G glycoprotein, inhibited viral fusion in vitro [110]. Despite encouraging results, human clinical data are lacking, and therapeutic benefit appears highly dependent on early administration, viral strain, and disease stage.

### 12.4. Immunotherapy and Monoclonal Antibodies

Monoclonal antibodies represent the most advanced targeted therapeutic approach for NiV infection. A fully human monoclonal antibody, m102.4, targeting the NiV G glycoprotein, has demonstrated potent neutralization in multiple animal models, including ferrets and non-human primates [111,112,113]. Phase I clinical trials confirmed that both single and repeated doses were safe and well tolerated, with no significant difference in adverse events compared to placebo [114]. Protection was time-dependent, with efficacy demonstrated when administered within days of exposure. m102.4 was also administered under compassionate use to 14 individuals with high-risk exposure to Hendra virus, demonstrating a favorable safety profile; however, controlled efficacy data in symptomatic NiV infection are lacking [115]. A humanized monoclonal antibody, h5B3.1, targeting the NiV F protein, inhibits viral membrane fusion by stabilizing the prefusion conformation and shows protection in ferret models. Further in vivo and clinical evaluation are required [96,107].

To reduce the risk of viral escape, combination monoclonal antibody (mAb) strategies are increasingly being considered for NiV infection. As with other RNA viruses, selective pressure from single-antibody therapy may promote the emergence of escape mutations, particularly when targeting a single epitope of the G glycoprotein. Combining mAbs targeting non-overlapping epitopes on the G and F proteins could enhance neutralization breadth and reduce the likelihood of resistance. Preclinical data suggest that antibody cocktails may provide more durable protection than monotherapy, and further in vivo evaluation is warranted to determine their therapeutic advantage and role in outbreak settings [88].

Currently, there are no approved antiviral therapies for NiV infection, and patient care is mainly based on supportive treatment; the therapeutic strategies for NiV are summarized in Table 1.

## 13. Prevention

### 13.1. Vaccines Against NiV

Nipah virus is classified as a high-priority pathogen by the World Health Organization due to its high case-fatality rate, zoonotic transmission, and pandemic potential. Despite extensive research efforts, no licensed vaccine for human use is currently available [65]. Vaccine development is challenged by the virus’s high pathogenicity, the sporadic and unpredictable nature of outbreaks, and logistical difficulties in conducting large-scale efficacy trials in endemic regions [95]. Nevertheless, substantial progress has been made in preclinical research, and several vaccine candidates have advanced to early-phase clinical evaluation. Multiple vaccine platforms are under investigation, including subunit vaccines targeting the G and F glycoproteins, viral vector vaccines (adenovirus, measles virus, recombinant vesicular stomatitis virus), mRNA vaccines, virus-like particle (VLP) vaccines, epitope-based and immunoinformatics-designed vaccines (Table 2).

### 13.2. Antigen Targets and Immunological Considerations

Current NiV vaccine development primarily targets the G and the F protein, which are essential for viral entry through interaction with ephrin B2/B3 receptors [110]. These glycoproteins are highly conserved between NiV and HeV, allowing the induction of cross-reactive immune responses. Effective vaccination is thought to require not only high titers of neutralizing antibodies but also a robust cellular immune response, including memory B and T cells, to ensure durable protection and potential control of reinfection [116,117].

### 13.3. Subunit Vaccines

Subunit vaccines represent the most advanced strategy in NiV vaccine development. A recombinant soluble G glycoprotein vaccine derived from HeV (HeV-sG-V; Equivac^®^ HeV) is currently licensed in Australia for use in horses to prevent HeV infection and reduce zoonotic transmission [118]. Importantly, this vaccine has demonstrated cross-protection against NiV in several animal models, including ferrets, cats, hamsters, and African green monkeys. In non-human primates, a single dose was sufficient to prevent lethal NiV challenge, while booster regimens provided long-term immunity in ferrets [119]. Although the vaccine is not effective in pigs, it is currently undergoing phase I clinical trials as a potential emergency vaccine for human NiV outbreaks [120].

### 13.4. Virus-like Particle Vaccines

Virus-like particle (VLP) vaccines mimic the structural organization of native virions while lacking viral genetic material, offering a favorable safety profile. VLPs incorporating NiV matrix (M), G, and F proteins were successfully generated in mammalian expression systems. These vaccines have demonstrated strong immunogenicity and protective efficacy in murine models, conferring protection against lethal NiV challenge after either single or multiple immunizations. Their ability to present conformational epitopes and induce both humoral and cellular immune responses makes VLP-based vaccines a promising platform, although they remain at the preclinical stage [121,122].

### 13.5. Recombinant Viral Vector Vaccines

A wide range of recombinant viral vector platforms has been explored for NiV vaccination, including vesicular stomatitis virus (VSV), measles virus, rabies virus, adenovirus, chimpanzee adenovirus, canarypox virus, cowpox virus, and Newcastle disease virus [123]. These vectors enable efficient expression of NiV surface glycoproteins and can induce strong humoral and cellular immune responses. Among these, VSV-based vaccines have demonstrated particularly strong efficacy, providing complete protection in hamsters, ferrets, and African green monkeys following single-dose immunization [124]. One such candidate, PHV02, which encodes the NiV G protein, has progressed to Phase I human clinical evaluation (study identifier NCT05178901 https://clinicaltrials.gov/study/NCT05178901?term=NCT05178901&rank=1 accessed on 2 March 2026). In nonhuman primate studies, vaccination with PHV02 resulted in complete protection against lethal NiV challenge, with survival strongly correlated with the presence of neutralizing antibodies prior to exposure [125].

Another promising candidate is the chimpanzee adenoviral-vectored candidate (ChAdOx1 NipahB), developed by the University of Oxford and supported by CEPI. This vaccine expresses NiV glycoprotein antigens and has entered first-in-human clinical evaluation in a Phase I trial involving 51 healthy adult participants, representing one of the most advanced NiV vaccine programs currently under development [80].

### 13.6. DNA and Inactivated Vaccines

DNA vaccines encoding NiV G or F proteins have demonstrated immunogenicity in animal models, with G-based constructs eliciting stronger neutralizing antibody responses. These vaccines offer advantages in terms of safety, stability and scalability, however they often require adjuvants or enhanced delivery systems, such as electroporation, to achieve sufficient immunogenicity. Inactivated NiV vaccines were investigated, primarily for antigen production and antibody generation, but their role in protective immunization remains limited [126,127].

### 13.7. mRNA Vaccines

The success of mRNA vaccine platforms against SARS-CoV-2 has accelerated their application to NiV. mRNA vaccines encoding NiV G or F proteins, delivered via lipid nanoparticles, showed strong immunogenicity and protective efficacy in preclinical animal models, including hamsters and non-human primates [128]. One candidate, mRNA-1215, entered phase I clinical trials in 2022 (study identifier NCT05398796 https://clinicaltrials.gov/study/NCT05398796 accessed on 2 March 2026) [129]. Although mRNA vaccines offer advantages such as rapid development and adaptability to viral mutations, further evaluation is required to determine the durability of long-term immunity and the feasibility of large-scale production.

### 13.8. Nanoparticle-Based Vaccines

Nanoparticle vaccines represent a highly promising strategy for NiV immunization. Ferritin-based self-assembling nanoparticles displaying NiV G protein domains have demonstrated superior immunogenicity compared with soluble antigens [130]. These vaccines induced rapid, broad, and durable neutralizing antibody responses and provided complete protection against lethal NiV challenge in hamster models. Importantly, they also elicited cross-neutralizing activity against both NiV lineages and Hendra virus, highlighting their potential for broad Henipavirus protection [131].

### 13.9. CD40-Targeted Vaccines

CD40-targeted vaccine strategies enhance antigen delivery to dendritic cells, promoting both humoral and cellular immunity through intrinsic adjuvant mechanisms [132,133].

A CD40-targeting Nipah virus vaccine (CD40.NiV), based on a humanized anti-CD40 monoclonal antibody fused with the NiV G glycoprotein and conserved epitopes from the F and nucleocapsid proteins, was recently evaluated in African green monkeys. Vaccination induced robust IgG, IgA, and neutralizing antibody responses, as well as T-cell immunity, and conferred complete protection against lethal NiV-B challenge with no detectable viral replication [134].

The enhanced immunogenicity of CD40-targeted vaccines is attributed to direct dendendritic cell targeting, which may function as an intrinsic adjuvant mechanism and promote early and durable immune responses [132]. The inclusion of conserved multi-epitope antigens may also provide cross-protection against related Henipaviruses [133]. However, despite these promising findings, CD40-based vaccine strategies remain at preclinical stage, and further studies are required to evaluate their safety, long-term immunogenicity, and potential translation into clinical applications.

### 13.10. Multi-Epitope/Immunoinformatics Vaccines

Recent advances in computational vaccinology have enabled the rational design of multi-epitope vaccines targeting conserved and immunogenic regions of the NiV proteome. Shabbir et al., (2025) [135] developed an immunoinformatics-driven multi-epitope vaccine candidate targeting the NiV nucleoprotein (N), a highly conserved antigen essential for viral replication and capable of eliciting strong B- and T-cell responses. Although immunoinformatics-based multi-epitope vaccines demonstrate promising predicted immunogenicity and favorable safety profiles in silico, these constructs remain at the preclinical design stage. Experimental validation in vitro and in vivo is required before progression to clinical evaluation [136].

## 14. Future Directions

Despite significant progress, several challenges remain in developing the NiV vaccine. The rarity and unpredictability of outbreaks complicate the conduct of phase III efficacy trials, while limited commercial incentives and resource constraints in endemic regions hinder large-scale clinical development [95]. Future efforts should focus on optimizing immunogenicity and durability, identifying immune correlates of protection, and integrating advanced technologies such as structural vaccinology, bioinformatics, artificial intelligence–guided antigen design, and innovative delivery systems. Strengthening international collaboration and adopting a One Health approach will be essential to advancing NiV vaccines from experimental stages to clinical and public health implementation [137,138].

## 15. Public Health Measures

The lack of targeted therapies and available vaccines renders both patient care and outbreak control more challenging. Raising public health awareness regarding NiV outbreaks, risk factors and the importance of preventive measures is crucial for self-protection and limiting transmission. Efforts to control transmission should initially target preventing bats from contaminating date palm sap and other fresh fruits through the use of protective coverings, avoiding raw sap consumption and cleaning and removing the skin of fruits prior to eating [65]. In areas susceptible to NiV, managing outbreaks requires high-level healthcare and comprehensive infection control strategies, including quarantine and the use of personal protective equipment (PPE) [88]. Close unprotected interactions with sick individuals should be minimized and hand hygiene should be performed consistently.

## 16. Research Priorities

Moore KA et al. [88] provided an update to the 2019 WHO Research and Development roadmap. Research priorities for 2024–29 regarding NiV should focus on accelerating the development of vaccines, therapeutics, and diagnostics to combat a pathogen with 40–100% fatality rates.

Diagnostics: The primary goal is to develop rapid, POC tests for immediate outbreak detection in remote or resource-limited areas. Achieving this goal requires a shift from centralized laboratory testing toward portable, high-accuracy diagnostic platforms that can be deployed directly in the field.

Therapeutics: In the absence of an approved treatment for NiV infection, research efforts are focused on evaluating promising antiviral and antibody-based therapies. Potential candidates include monoclonal antibodies such as m102.4 monoclonal antibody and small-molecule antivirals like Remdesivir, with further investigation needed through Phase I and II clinical trials conducted in regions at risk of outbreaks.

Vaccine Development: As there is no vaccine currently approved for use against Nipah virus, researchers should prioritize their development and their entrance into clinical trials to ensure their safety and immunogenicity [88].

## 17. Conclusions

Nipah virus represents a challenging zoonotic threat characterized by high fatality rates and periodic re-emergence. Its dual manifestation as acute encephalitis and severe respiratory distress, alongside its capacity for interpersonal transmission, underscores its status as a high-priority pathogen with global pandemic risks. Although advances in diagnostics, experimental therapies, and vaccine research are promising, no approved treatment or human vaccine is currently available. Strengthened surveillance, infection control, and a coordinated One Health approach are essential to prevent spillover events and improve global preparedness against future outbreaks.

## Figures and Tables

**Table 1:** Therapeutic Strategies for NiV Infection.

Agent	Mechanism of Action	Evidence Level	Key Findings	Limitations	References
Ribavirin	Broad-spectrum RNA synthesis inhibitor	Human outbreak data (open-label); animal studies; compassionate use	36% mortality reduction reported in 1998–1999 Malaysia outbreak; 20% mortality reduction in 2018 Kerala outbreak	Non-randomized; historical controls; no proven survival benefit in animal models; controversial efficacy	Chong et al., 2001 [102]; Aljofan et al., 2009 [101]; Georges-Courbot et al., 2006 [109]; Chandni et al., 2020 [66]
Acyclovir	DNA polymerase inhibitor	Empirical use only	Used during Singapore outbreak; favorable outcomes reported	No in vitro or animal evidence against NiV; unclear contribution	Goh et al., 2000 [63]
Remdesivir (GS-5734)	RNA-dependent RNA polymerase inhibitor	Non-human primate (African green monkeys)	100% survival when IV treatment initiated within 24 h of exposure (12-day course)	No human NiV data; efficacy unproven after symptom onset; IV administration required	Lo et al., 2019 [106]
Favipiravir	RNA polymerase inhibitor	Hamster model	Complete protection when administered immediately post-infection	No NHP or human NiV data; narrow treatment window	Dawes et al., 2018 [107]
Balapiravir (R1479)	Nucleoside polymerase inhibitor	In vitro	Inhibits NiV and HeV replication	Poor bioavailability; prior toxicity concerns; no advanced development	Hotard et al., 2017 [108]
Soluble Ephrin-B2	Blocks viral G protein receptor binding	In vitro	Inhibits NiV/HeV binding to target cells	No animal or human therapeutic data	Negrete et al., 2005 [110]
Poly(I)-poly(C12U)	Stimulates innate immune response	Animal model	Partial protection in preclinical studies	Limited efficacy; no human data	Georges-Courbot et al., 2006 [109]
m102.4	Neutralizes NiV G glycoprotein; blocks receptor binding	NHP models + Phase I human safety data	Protection in ferrets and NHPs; safe and well tolerated in Phase I; used in 14 compassionate-use cases	Time-dependent efficacy; no controlled efficacy data in symptomatic NiV	Bossart, K.N. et al., 2009 [111]
h5B3.1	Targets NiV F protein; inhibits membrane fusion	Ferret model	Post-exposure protection in animal studies	No human data; early development stage	Mire, C.E. et al., 2020 [96]
Antibody cocktails	Target non-overlapping G and F epitopes	Preclinical concept	Potential reduction in viral escape; broader neutralization	Not yet clinically evaluated	Moore, K.A. et al., 2024 [88]

Abbreviations: NiV: Nipah Virus, IV: intravenous, NHP: Non-Human Primates, Hev: Hendra Virus, RNA: ribonucleic acid, DNA: deoxyribonucleic acid, m102.4: human monoclonal antibody m102.4, h5B3.1: Human monoclonal antibody h5B3.1, G protein: attachment glycoprotein, F protein: fusion protein.

**Table 2:** NiV Vaccine Candidates.

Platform	Key Candidate(s)	Target Antigen	Stage/Notes
Subunit Vaccines	HeV-sG-V (Equivac^®^ HeV)	G glycoprotein (HeV/NiV cross-reactive)	Licensed in horses; phase I human trials for emergency NiV use; cross-protective in ferrets, hamsters, African green monkeys
VLP Vaccines	NiV VLPs	M, G, F proteins	Preclinical; strong immunogenicity and protection in mice
Recombinant Viral Vector Vaccines	VSV-vectored PHV02; Measles virus vector, ChAdOx1 NiVB	G glycoprotein	Preclinical efficacy in hamsters, ferrets, NHP; PHV02 in phase I human trials Phase I human trial with 51 participants (first-in-human)
DNA Vaccines	NiV G/F constructs	G/F proteins	Preclinical; G-based constructs elicit stronger neutralizing antibodies; may require adjuvants or electroporation
mRNA Vaccines	mRNA-1215	G/F proteins	Preclinical: protective in hamsters and NHP; Phase I trials initiated 2022
Nanoparticle-Based Vaccines	Ferritin-based NiV G nanoparticles	G protein domains	Preclinical; induce broad, durable neutralizing antibodies; cross-neutralize NiV lineages and HeV
CD 40 targeted vaccines	CD40.NiV	G glycoprotein with conserved F and N epitopes	Preclinical; induced humoral and cellular responses, cross-neutralization, and complete protection in African green monkeys
Multi-Epitope/Immunoinformatics Vaccines	Designed via computational modeling	Multiple B/T cell epitopes, nucleoprotein	Preclinical; high predicted immunogenicity, population coverage; requires experimental validation

Abbreviations: HeV: Hendra Virus, NiV: Nipah Virus, HeV-sG-V: Hendra virus soluble G glycoprotein vaccine, VLP: Virus-Like Particle, VSV: Vesicular Stomatitis Virus, ChAdOx1: Chimpanzee Adenovirus Oxford 1, NiVB: Nipah Virus Bangladesh strain, DNA: Deoxyribonucleic acid, mRNA: messenger RNA, NHP: Non-Human Primates, G protein: attachment glycoprotein, M: matrix protein, F protein: fusion protein.

## Data Availability

Data are contained within the article.

## Abbreviations

The following abbreviations are used in this manuscript:

NiV	Nipah Virus
CFR	Case Fatality Rate
WHO	World Health Organization
CDC	Centers for Disease Control
NIAID	Prevention and the National Institute of Allergy and Infectious Diseases
JEV	Japanese encephalitis virus
NiV-MY	Nipah Virus Malaysian strain
NiV-BD	Nipah Virus Bangladesh strain
Hev	Hendra Virus
N	nucleocapsid protein
P	phosphoprotein
M	matrix protein
F	fusion protein
G	attachment glycoprotein
L	large polymerase
vRNP	virus ribonucleoprotein
ER	endoplasmic reticulum
IL-	interleukin-
G-CSF	granulocyte colony-stimulating factor
PCR	polymerase chain reaction
RT-PCR	Reverse Transcription-Polymerase Chain Reaction
qRT-PCR	Quantitative Reverse Transcription PCR
CSF	Cerebrospinal Fluid
ARDS	Acute Respiratory Distress Syndrome
MODS	Multiple Organ Dysfunction Syndrome
HSV	Herpes Simplex Virus
ELISA	Enzyme-Linked Immunosorbent Assay
BSL-	Biosafety level-
ELISA	ELISA: Enzyme Linked Immuno Sorbent Assay
POC	Point of Care
LFA	Lateral flow assays
VLP	Virus-like particle
VSV	Vesicular Stomatitis Virus
PPE	Personal Protective Equipment
